# A case of total scaphoid titanium custom-made 3D-printed prostheses with one-year follow-up

**DOI:** 10.1080/23320885.2019.1708203

**Published:** 2020-01-08

**Authors:** Mario Igor Rossello

**Affiliations:** Hand Surgery Department “Renzo Mantero”, Ospedale San Paolo, Savona, Italy

**Keywords:** 3D-printed implant, carpal bones, degenerative arthritis, nonunion, scaphoid fracture, scaphoid nonunion advanced collapse arthritis, Silastic

## Abstract

A 34-year-old male patient developed scaphoid nonunion with necrosis in both fragments. A scaphoid replacement was performed using a titanium custom-made 3D-printed implant. At 1-year follow-up, good clinical and radiographic outcome was obtained. Titanium custom-made 3D-printed implants may offer a good surgical solution for patients requiring total scaphoid replacement.

## Introduction

Sequelae of scaphoid fracture treatment include nonunion and necrosis of bone fragments. Carpal biomechanical changes can lead to scaphoid nonunion advanced collapse (SNAC) arthritis and severe degenerative osteoarthritis of radiocarpal and intercarpal joints. Surgical treatment options for the reconstruction of the scaphoid bone or, when that is impossible, for the prevention or treatment of late sequelae, include bone grafts (free or pedicled when the nonunion is isolated without collapse phenomena) [1–7], proximal row carpectomy [7,8], total or partial scaphoid removal in association with midcarpal arthrodesis, total wrist replacement, and total wrist arthrodesis [9]. However, no one method is considered to be the gold standard.

The first scaphoid prosthesis was constructed from vitallium in 1945 [10], followed by the acrylic implant which was designed in 1950 [11]. However, in 1962, a truly revolutionary implant – the Silastic™ (Dow Corning Corporation, Midland, MI, USA) prosthesis – was introduced by Swanson [7,12–15]. The implant was increasingly used globally until severe problems of silicone synovitis were observed on long-term follow-up. However, biomechanical or anatomical problems were not reported for this implant [16]. The only disadvantages cited were silicone synovitis and severe wrist destruction caused by particulate implant debris [17]. Subsequently, Swanson developed the titanium implant in 1989. This prosthesis averted siliconitis while maintaining the good biomechanical and anatomic results of the silicone scaphoid implant [16]. However, the titanium implant did not attain the popularity of the silastic implant.

When clinical evaluation of scaphoid necrosis reveals scaphoid destruction that is unsuitable for grafting, good wrist stability without a SNAC wrist condition (confirmed by carpal height and radiolunate angle measurements), and no degenerative changes in the radial scaphoid facet and/or other carpal bones, then scaphoid total replacement should be considered [18,19].

Once the radiographic examination is completed, an exact diagnosis is confirmed through magnetic resonance imaging and computed tomography (CT). These images provide more information for the assessment of nonunions with displaced fragments, residual malunion, and bone necrosis.

Scaphoid replacement should not be performed in those patients with radial scaphoid facet degeneration, prior radial styloidectomy, carpal collapse and deformity of the distal radius following displaced fracture, diminished McMurtry index, increased radiolunate angle [7], or degenerative arthritis of carpal bones especially at the midcarpal joint.

Total scaphoid prosthetic replacement is reserved for cases where carpal collapse and signs of wrist instability are not observed, and where proximal row resection, midcarpal arthrodesis and wrist replacement are not indicated. If scaphoid nonunion without important bone reabsorption is observed, bone grafts are preferred. Partial replacement is considered when nonunion has led to isolated aseptic necrosis of the proximal pole of the scaphoid.

Recently, a new generation of custom-made prostheses using 3D printing technology was introduced [20]. These implants are made of titanium alloy employing an electron beam melting (EBM) additive manufacturing process. 3D printing technology may provide the surgeon with an additional option for the treatment of scaphoid nonunion.

## Case report

**Figure 1.**
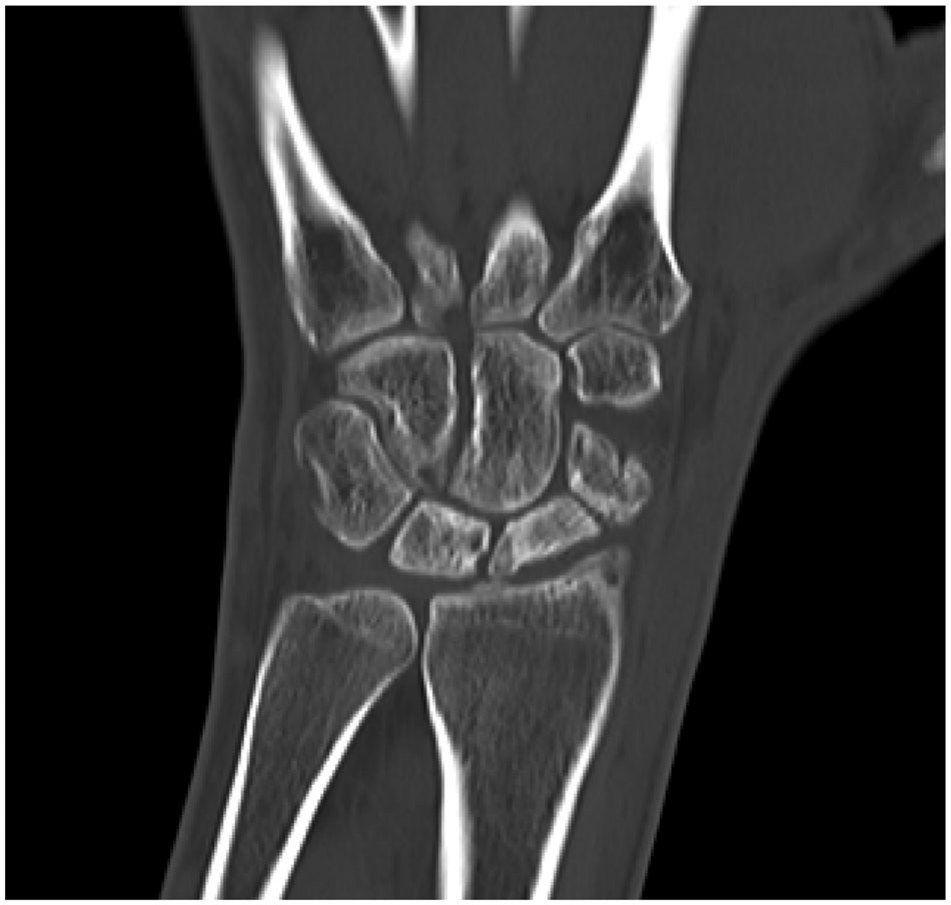
Preoperative computed tomography scan image showed scaphoid nonunion with wide necrosis areas with no evidence of radiocarpal arthritis or scaphoid nonunion advanced collapse wrist.

We report the case of a 34-year-old male who experienced wrist trauma three years ago. The patient ignored the injury until severe pain and functional loss appeared over a six-month period. A CT scan demonstrated a scaphoid nonunion that occurred some time ago with evident signs of necrosis in both fragments (Figure 1). There were no signs of significant changes in the other carpal bones, and neither was advanced SNAC observed.

After a review of the different surgical options (total scaphoid implant, proximal row carpectomy, 3- or 4-corner arthrodesis, or no surgery but completion of wrist denervation), the patient agreed to undergo a total scaphoid replacement with a custom-made 3D implant (Adler Ortho, Cormano, Italy). The implant had a titanium niobium nitride (TiNbN) coating to reduce its allergic potential and to improve its surface hardness. The surgical technique consisted of a dorsal approach between the extensor carpi radialis brevis and extensor pollicis longus. The wrist capsule with the radiocarpal dorsal ligament was cut in a T-shape with dissection from the radius and carpal bones so that the necrotic scaphoid was exposed and isolated.

**Figure 2.**
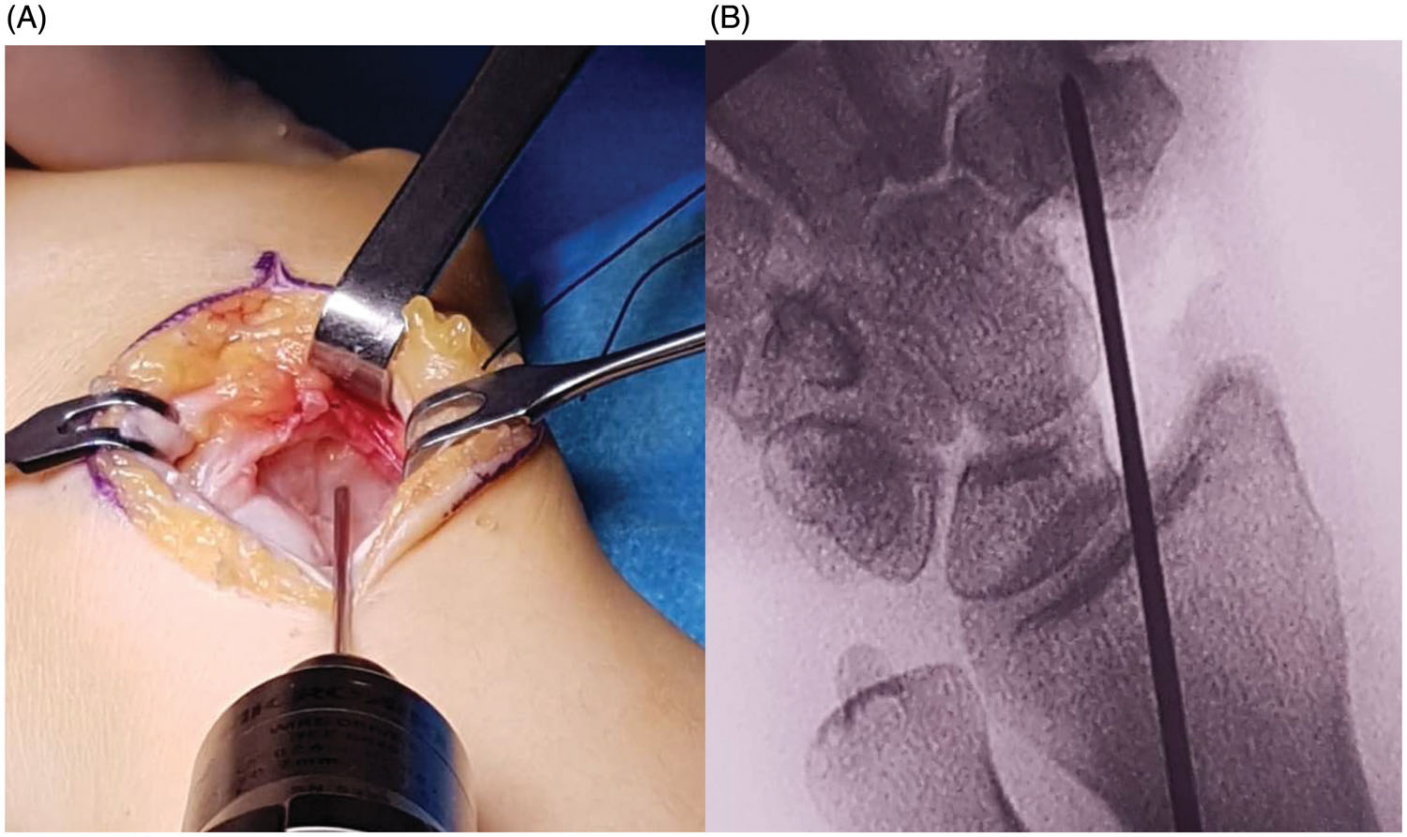
(A,B) A hole was placed into the trapezium to host the distal carpal stem.

**Figure 3.**
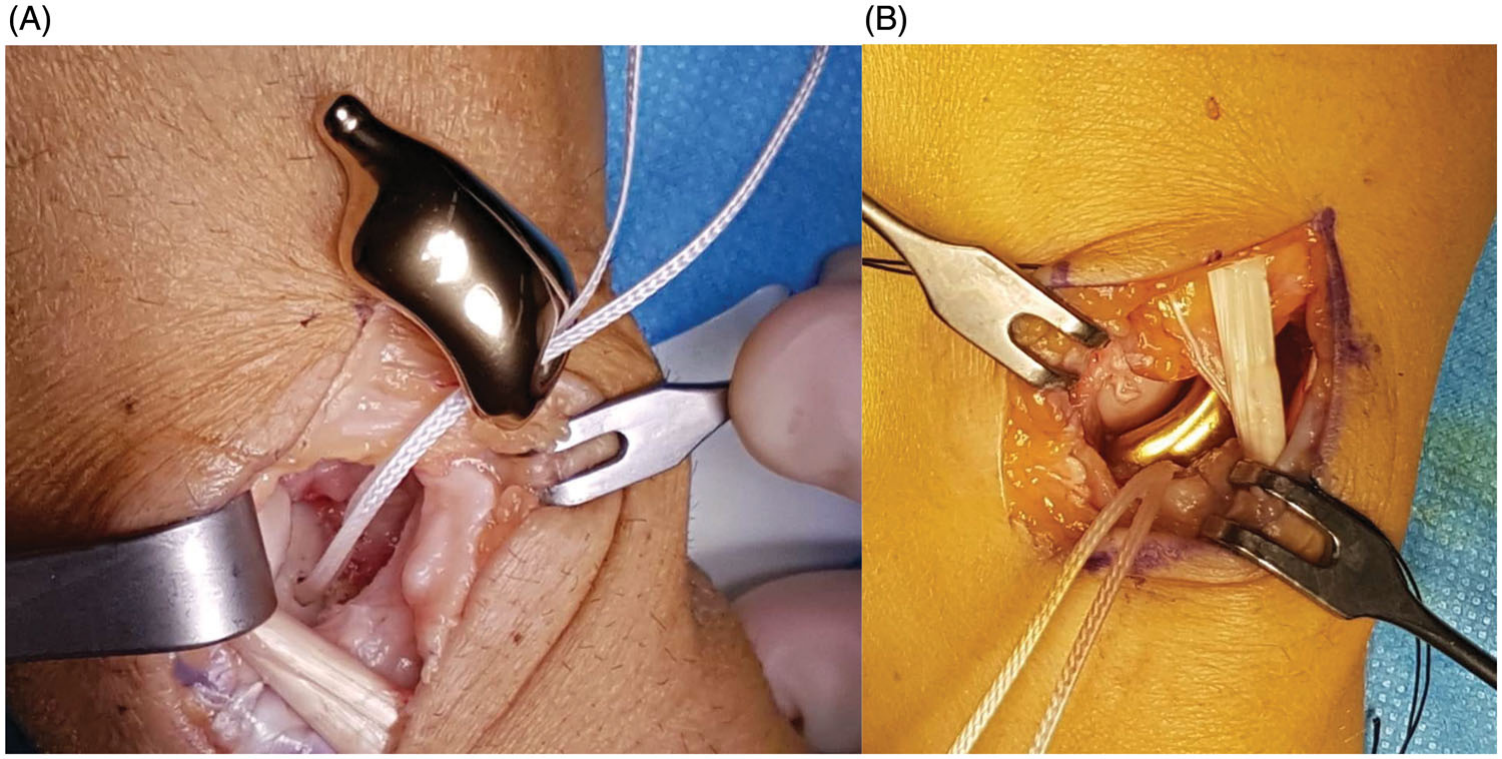
(A,B) The titanium implant was positioned. Arthrex™ labral tape was fixed into the lunate and passed through the implant. The distal stem of the implant was set into the hole prepared into the trapezium.

**Figure 4.**
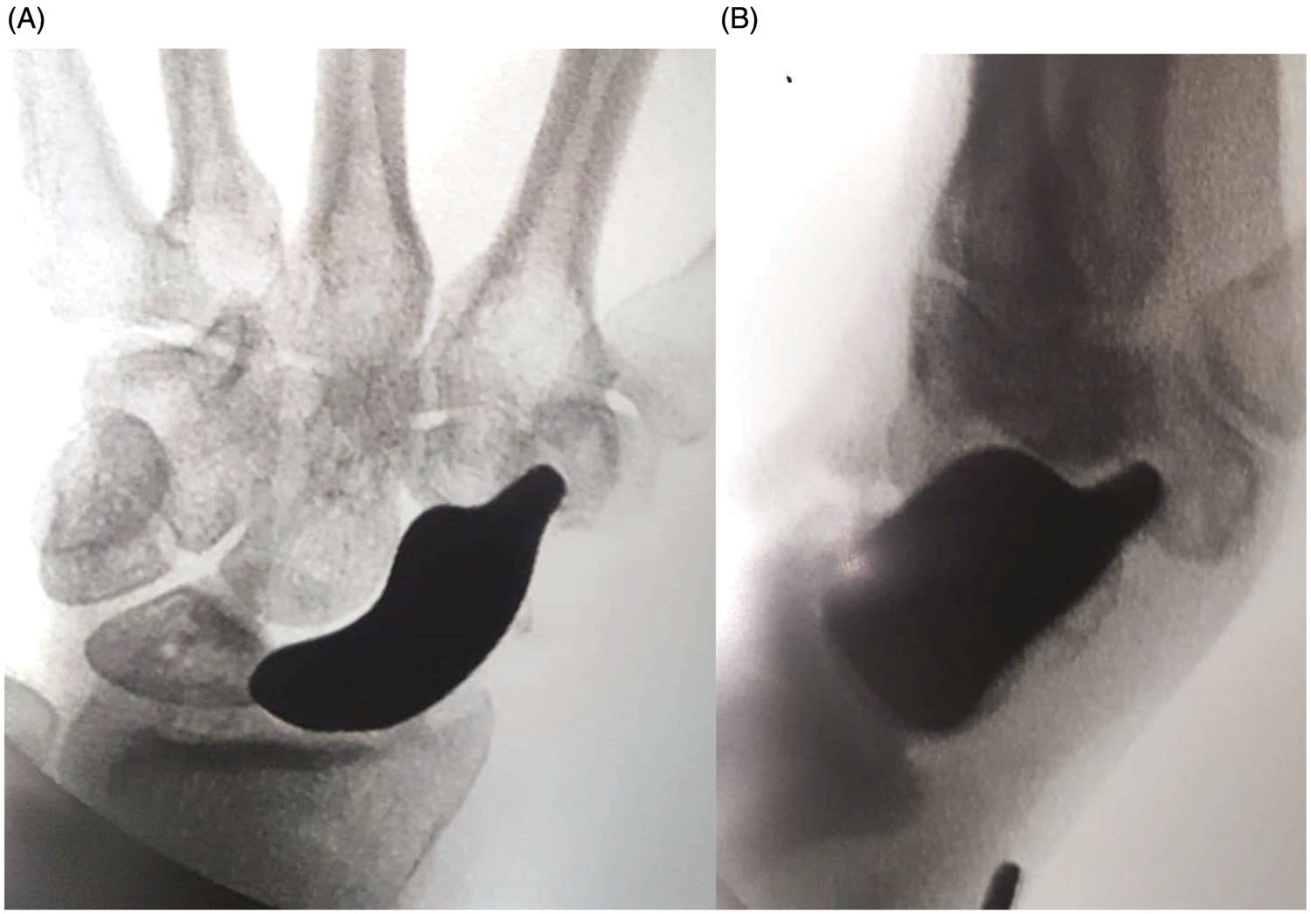
(A,B) In the perioperative radiography, the prosthesis position was verified as well as the position of the distal stem.

After removing the necrotic tissue (while leaving a small volar part of the distal pole to preserve the radioscaphocapitate ligament insertion), a hole was made in the trapezium under image amplifier control (Figure 2). This hole was designed to host the distal tip of the prosthesis, a key point to stabilize the implant. To reconstruct the scapholunate ligament, Arthrex™ labral tape (Naples, FL, USA) was inserted into the lunate with an anchor. The implant was then prepared for positioning. First, the two cords of the labral tape were passed through the corresponding holes of the implant, and then the prosthetic distal tip was inserted into the trapezium hole. The implant was fitted into its space, and the two labral tape cords were finally knotted in an apposite notch carved into the implant (Figure 3). The implant was therefore stabilized on both sides: distally by its tip inserted into the trapezium and proximally thanks to the labral wire fixed to the lunate. After confirming a satisfactory position radiographically (Figure 4), implant stability was tested by moving the wrist in any direction. The dorsal capsule was closed and a suction drain was left in place for 24 hrs. The entire surgery lasted 45 min.

On the first postoperative day, the dressing was changed and a thermoplastic splint was applied, maintaining the wrist in alignment with the forearm with a 10° extension. Immobilization was sustained for 4 weeks, and then a radiographic examination was performed to confirm the satisfactory position of the implant. Subsequent rehabilitation was then conducted for 3 months.

**Figure 5.**
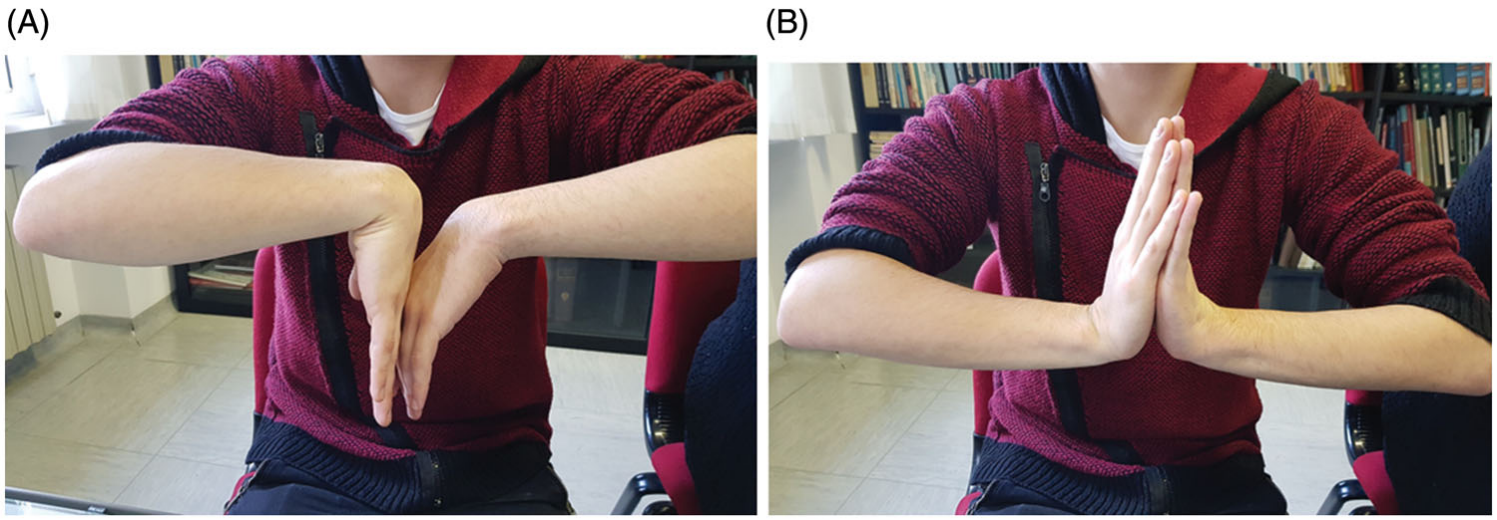
(A,B) This image displayed range of motion after 1 year.

**Figure 6.**
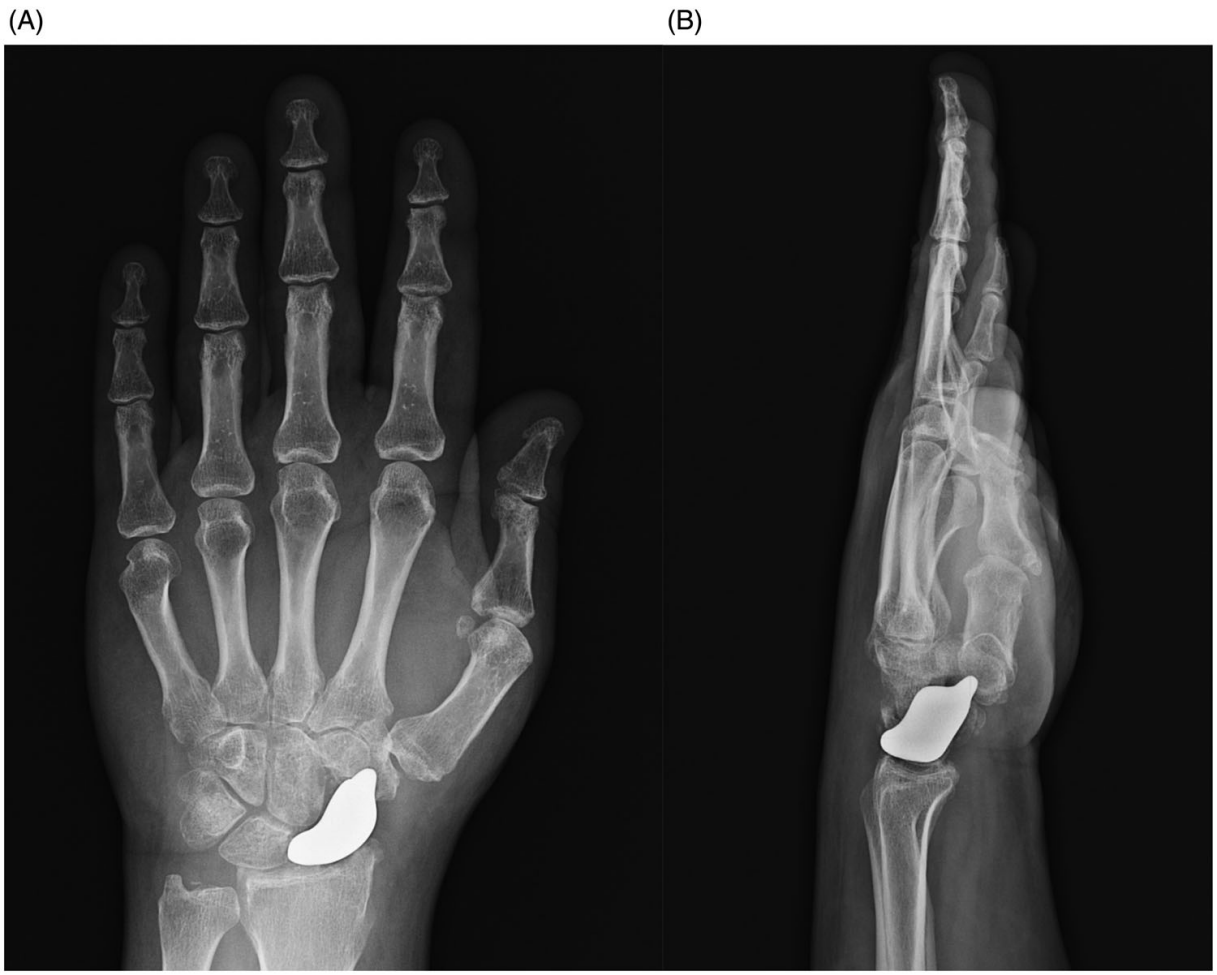
(A,B) At 1-year follow-up, radiography showed a correct radioscaphoid angle, with no scapholunate gap and good reconstruction of Gilula Arcs with entire near-to-normal carpal anatomy.

Clinical and radiographic assessments were performed at 1, 3, 6, and 12 months after surgery. At the final follow-up, significant improvement in terms of pain and function was observed (Tables 1 and 2, Figure 5). Furthermore, radiography demonstrated a near-to-normal restoration of carpal biomechanics without the evolution of wrist instability or any changes in the carpal bones (Figure 6).

**Table 1.** Clinical outcome of patient with titanium custom-made 3D-printed implant.

	Contralateral	Preoperative	1 Year postoperative
Active ROM*			
Extension	80°	20°	70°
Flexion	90°	45°	70°
Radial deviation	35°	15°	30°
Ulnar deviation	35°	35°	35°
Prono-supination	Complete	Complete	Complete
Strength (Jamar)			
Grip	48 Kgm	24 Kgm	30 Kgm
Pinch	9 Kgm	3 Kgm	9 Kgm
Pain VAS^†^ scale			
At rest	0	3	0
Under load	0	6	1

^*^ ROM: range of motion; ^†^VAS: visual analogue scale.

**Table 2.** Preoperative and postoperative assessment of anatomical function of titanium custom-made 3D-printed implant.

DASH* score	Preoperative	1 Year postoperative
General	43.3	13.3
Work	68.8	12.5
Sport/Music	75	12.5
PRWE^†^	53	11

^*^ DASH: disabilities of the arm, shoulder and hand; ^†^PRWE: patient-rated wrist evaluation.

Written informed consent was obtained from the patient for publication of this case report.

## Discussion

A total scaphoid implant should be considered in patients with specific indications. The main advantage of the technique is the restoration of wrist anatomy with close-to-normal biomechanics. Furthermore, only the affected bone is involved in the surgery. Additionally, in case of failure, alternative procedures, such as traditional scaphoidectomy, 3- or 4-corner arthrodesis, or proximal row carpectomy are possible.

Strict attention must be given to ensure a correct preoperative indication and implant size, as well as to provide a precise technical execution that preserves the prosthetic stability of the volar and dorsal ligaments and distal stem. The prosthetic stem has the same effect as a scapho-trapezium-trapezoid arthrodesis, in which the loads shift from the radioscaphoid joint to the scapho-trapezium-trapezoid complex. Further stability is provided through the radioscaphocapitate ligament (volarly), and the scaphotrapezium ligament (dorsally). The scapholunate interosseous ligament is reconstructed with the labral tape inserted into the lunate and knotted to the implant. The volar ligament is preserved via the dorsal approach, and the dorsal ligament must be reconstructed after prosthesis positioning.

After 28 years of positive results with the Swanson implant, a new concept based on 3D printing technology is now available. The advantage of 3D-printed prosthetics is that it provides full anatomical reconstruction [20]. The challenge of reproducing the patient’s own morphology is being solved. The 3D model is also useful to assess the implant fit preoperatively. The surgeon is able to accurately plan the surgical procedure with the use of the 3D model, thereby reducing surgical time.

Furthermore, unline the conventional silicone-made model, the presented implant has a system for scapholunate ligament reconstruction. TiNbN coated titanium alloy also has excellent biocompatibility. We believe these features jointly will be able to mitigate the main disadvantages of the Swanson silicone implant: instability of the prosthesis and silicone-induced synovitis [21].

When compared to standard procedures, 3D printing technology allows for a major simplification of the design and manufacturing process of implants. However, the whole process still takes about 3 weeks [20]. The manufacturing cost is another important factor to consider, and the technology’s cost-effectiveness is yet to be established. While further follow-up is needed, titanium custom-made 3D-printed implants may offer a good surgical solution for patients requiring total scaphoid replacement.
